# Evaluation of community pharmacists’ knowledge and real-world practices in drug interaction management: a cross-sectional study

**DOI:** 10.3389/fmed.2026.1771828

**Published:** 2026-04-15

**Authors:** Abdulaziz F. Alhazmi, Firas A. Alghuthmi, Mohammed A. Hamoh, Khalid A. Almasari, Abdulmajeed M. Zagzoug, Moayed M. Kenkar, Saeed M. Tayeb, Mohammed M. Aldurdunji, Eman O. Alsheikh, Ohood K. Almuzaini, Saad M. Wali

**Affiliations:** 1 College of Pharmacy, Umm Al-Qura University, Makkah, Saudi Arabia; 2 Pharmaceutical Sciences Department, College of Pharmacy, Umm Al-Qura University, Makkah, Saudi Arabia; 3 Pharmaceutical Practices Department, College of Pharmacy, Umm Al-Qura University, Makkah, Saudi Arabia; 4 Department of Pharmacology and Toxicology, Faculty of Medicine, Umm Al-Qura University, Al-Qunfudhah, Makkah, Saudi Arabia; 5 Pharmacology and Toxicology Department, College of Pharmacy, Umm Al-Qura University, Makkah, Saudi Arabia

**Keywords:** clinical practice, community pharmacy, drug–drug interactions, patient safety, pharmacist performance, Saudi Arabia, simulated patients

## Abstract

**Introduction:**

Drug–drug interactions (DDIs) remain a significant but often overlooked threat to patient safety. While Pharmacists are expected to play a critical role in identifying and preventing DDIs, real-world practice may not reflect this responsibility. This study aimed to assess the gap between community Pharmacists’ DDI knowledge and their actual dispensing practices in Western Saudi Arabia.

**Methods:**

A sequential explanatory mixed-methods study was conducted between September and November 2025 among 356 licensed community Pharmacists in Makkah, Jeddah, Madinah, and Taif. The quantitative phase involved a validated, structured questionnaire evaluating Pharmacists’ knowledge and self-reported practices regarding DDIs. This was followed by a qualitative observational phase involving 134 unannounced simulated patient (SP) visits, each presenting one of four predefined high-risk DDI scenarios (ibuprofen–furosemide, omeprazole–clopidogrel, fluconazole–phenytoin, and phenytoin–warfarin). Statistical analyses included descriptive statistics, non-parametric group comparisons (Wilcoxon and Kruskal–Wallis tests), multivariable linear regression, and Fisher’s exact test, conducted using RStudio (*p* < 0.05).

**Results:**

Survey findings demonstrated moderate to high theoretical DDI awareness, with 62–79% of Pharmacists correctly identifying major interaction pairs. Pharm. D. graduates achieved higher knowledge scores than B. Pharm graduates (median 5.0 vs. 4.0; *p* < 0.001). However, simulated visits revealed a substantial practice gap: only 15% of Pharmacists identified the DDI in real-time, and approximately 75% took no corrective action. Most encounters lasted less than one minute, and over 80% of interacting medications were dispensed without inquiry or reference checking.

**Conclusion:**

Despite adequate theoretical knowledge, community Pharmacists demonstrated limited real-world application of DDI management. This knowledge–practice gap poses a patient safety concern and underscores the need for system-level interventions, including decision-support tools, workflow optimization, and strengthened prescriber–Pharmacist communication.

## 1 Introduction

Drug–drug interactions (DDIs) represent a critical yet often overlooked threat to patient safety. These interactions occur when the pharmacological or clinical effect of one drug is altered by the presence of another—leading to diminished therapeutic efficacy, increased toxicity, or unexpected adverse reactions. Globally, DDIs account for approximately 20–30% of all adverse drug events, making them a significant source of preventable harm in clinical practice (1–3). Beyond the health burden, the economic implications are substantial, contributing to avoidable hospitalizations, prolonged treatment durations, and increased healthcare expenditure.

Community Pharmacists occupy a pivotal role in the healthcare delivery chain. As the final healthcare professionals to interact with patients before medication use, they serve as a crucial checkpoint in ensuring medication safety. However, unlike hospital Pharmacists, community Pharmacists often operate without access to complete patient histories, laboratory data, or integrated clinical support systems. This necessitates rapid decision-making, where Pharmacists must evaluate prescriptions on the spot, recognize potential DDIs, and intervene appropriately. Their role extends beyond dispensing, public expectations include proactive vigilance and clinical judgment under pressure (4, 5).

Despite strong expectations, a persistent gap exists between theoretical knowledge and clinical behavior. Prior studies have demonstrated that Pharmacists frequently perform well in DDI knowledge assessments (6). However, this proficiency does not consistently translate into real-world action. Observational and simulated patient studies have highlighted a concerning trend: Pharmacists often fail to detect or respond to DDIs during everyday practice. The discrepancy between what professionals know and how they act in routine scenarios warrants further investigation (7).

International evidence has documented similar discrepancies between community Pharmacists’ knowledge of DDIs and their real-world dispensing practices. Studies conducted in Europe, Asia, and the Middle East have reported moderate to high awareness of clinically significant DDIs, yet consistently low rates of active screening, documentation, and intervention during routine dispensing (7–10). These findings suggest that knowledge alone may be insufficient to ensure safe DDI management in community pharmacy practice.

In Saudi Arabia, the community pharmacy sector presents a unique context for exploring this challenge. Pharmacies cater to a diverse population, including millions of international pilgrims visiting for Hajj and Umrah, many of whom have chronic illnesses, incomplete medication records, and face language barriers (11, 12). The system is rapidly evolving, with efforts toward digitalization and an increasingly diverse workforce comprising both Bachelor of Pharmacy (B. Pharm) and Doctor of Pharmacy (Pharm. D) graduates. Whether this evolving education landscape has improved DDI management practices remains unclear.

Existing literature in Saudi Arabia has largely focused on self-reported survey data to assess Pharmacists’ DDI knowledge (13, 14). However, such methodologies are prone to social desirability bias, where participants report ideal rather than actual behaviors. Regional studies have similarly reported variable knowledge levels and limited pharmacist-led interventions, highlighting the need for practice-based evaluation approaches in Saudi community pharmacies (15, 16).

Simulated patient (SP) methodology offers a more objective and reliable alternative. By using trained individuals to present realistic clinical scenarios covertly, researchers can observe authentic behavior without interference (17–19). This gold-standard method has been widely adopted internationally but remains underutilized in Saudi community pharmacy research.

Few studies in Saudi Arabia have integrated both survey-based and simulated patient methodologies to comprehensively assess DDI management in community pharmacy settings. Therefore, this study aims to provide a comprehensive, practice-based evaluation of DDI management in community pharmacies across Western Saudi Arabia. Specifically, it sought to assess Pharmacists’ knowledge using a validated survey, observe their real-world behavior through unannounced simulated patient visits, identify factors associated with higher knowledge and intervention rates, and quantify the gap between theoretical knowledge and actual clinical practice.

## 2 Methods

### 2.1 Study design

The study followed a sequential explanatory mixed-methods design, in which the quantitative survey phase was conducted first, from September to mid-October 2025, followed by the simulated patient (SP) phase from mid-October to the end of November 2025. An interval was maintained between phases to minimize recall bias and reduce the likelihood that pharmacists would anticipate simulated visits, thereby preserving the validity of observed practice behaviors.

This cross-sectional study combined a structured knowledge survey with unannounced simulated patient encounters and was conducted between September and November 2025 in community pharmacies across four major cities in Western Saudi Arabia: Makkah, Jeddah, Madinah, and Taif. These cities were purposively selected to reflect diverse practice settings—Makkah and Madinah serve millions of international pilgrims annually, Jeddah represents a major urban and commercial hub, and Taif reflects a smaller city with distinct geographical and healthcare characteristics. Only licensed retail community pharmacies were included. Hospital pharmacies, pharmacy students, and unlicensed personnel were excluded to ensure that findings reflected routine community pharmacy practice. The study design, conduct, and reporting adhered to the STROBE (Strengthening the Reporting of Observational Studies in Epidemiology) guidelines for observational research.

### 2.2 Study setting and participants

Licensed community Pharmacists were recruited through professional networks, local pharmacy associations, and direct outreach visits. Participation was entirely voluntary and anonymous. A convenience sampling strategy was used. An *a priori* sample size calculation was performed using G*Power (version 3.1) for multiple linear regression (fixed model, single regression coefficient), assuming a two-sided significance level of 0.05, 80% statistical power, a moderate effect size (f^2^ = 0.15), and five predictors. The calculation indicated a minimum required sample size of 55 participants. However, to ensure greater precision and stability of the regression estimates, and to account for potential variability and the exploratory nature of the analyses, a substantially larger sample size was targeted. The final sample (*n* = 356) exceeded this minimum requirement.

For the SP phase, the study aimed to conduct around 30 visits for each drug–drug interaction (DDI) scenario, totaling between 120 to 150 visits. Whenever feasible, SP visits were carried out in the same pharmacies where the Pharmacists had previously completed the survey, allowing for within-subject comparison between self-reported knowledge and actual observed behavior.

### 2.3 Survey instrument

The structured questionnaire used in the first phase was adapted from previously validated tools used in studies assessing Pharmacists’ knowledge of DDIs in Saudi Arabia (13). It consisted of two main sections. The first assessed Pharmacists’ knowledge of common and clinically relevant drug interactions using multiple-choice questions. The second section focused on daily practice, including how often Pharmacists encounter DDIs, their management strategies, and the reference tools they commonly use.

The questionnaire was pilot tested among 35 licensed community Pharmacists to assess clarity, cultural relevance, and practical feasibility. Based on pilot feedback, minor modifications were made, including rephrasing ambiguous items, simplifying technical terminology, and refining response options to improve comprehension and consistency. The final survey was then distributed through professional networks, local pharmacy associations, and direct outreach visits, and all responses were collected anonymously.

### 2.4 Simulated patient (SP) visits

In the second phase, trained data collectors conducted simulated patient visits in the same four cities. A total of eight data collectors participated, with two assigned to each city. All data collectors had prior research-related experience and received standardized training before study implementation. Training included detailed orientation on the study objectives, familiarization with the simulated patient methodology, scenario scripts, expected responses to pharmacists’ questions, and role-play rehearsals to ensure consistency across visits.

Each simulated patient presented one of four predefined and standardized clinical scenarios involving commonly encountered, high-risk DDI pairs: ibuprofen with furosemide, omeprazole with clopidogrel, fluconazole with phenytoin, and phenytoin with warfarin. These scenarios were developed based on their clinical relevance, frequency in community pharmacy practice, and pharmacological diversity, and were reviewed by experts in clinical pharmacy to ensure content validity (13, 20, 21).

Prior to formal data collection, the scenarios were piloted in community pharmacies not included in the final sample to assess feasibility, clarity, and realism. Minor refinements were made to scenario wording and response flow based on pilot feedback. For each scenario, standardized response scripts were developed to guide how simulated patients would respond to pharmacists’ questions regarding medication use, dosing, duration, and symptoms. These scripts were applied uniformly across all cities to minimize inter-simulated patient variability.

During each visit, the simulated patient requested both medications without indicating awareness of a potential interaction. Immediately after the encounter, the data collector completed a structured checklist developed by the research team, based on guideline-recommended practices for drug–drug interaction (DDI) management and published simulated patient studies. The checklist was reviewed and approved by experts in clinical pharmacy to ensure content validity. It consisted primarily of binary (Yes/No) and categorical items capturing key pharmacist responses, including whether a prescription was requested, whether the DDI was recognized, whether counseling or clinical intervention was provided, whether reference tools were consulted, and whether the interacting medications were dispensed together. To minimize recall bias, data collectors completed the checklist immediately after exiting the pharmacy using a secure online data collection form. Review of outcomes across cities revealed no systematic differences in pharmacist responses for identical scenarios, supporting consistency in simulated patient delivery. Statistically, internal consistency analysis of selected simulated patient responses demonstrated excellent reliability (Cronbach’s alpha = 0.955), further supporting the consistency of data collection.

### 2.5 Ethics approval and consent to participate

This study was approved by the Scientific Biomedical Research Ethics Committee at the College of Medicine, Umm Al-Qura University with approval number: HAPO-02-K-012-2025-09-2876. The participants provided digital written informed consent. The study was conducted in accordance with the ethical principles outlined in the Declaration of Helsinki. All participants were informed about the purpose of the study, assured of confidentiality, and provided written consent prior to participation. Participation was voluntary, and respondents could withdraw at any time without consequence.

### 2.6 Statistical analysis

All statistical analyses were conducted using R (version 4.4.2) within RStudio (version 2024.9.1.394). Descriptive statistics summarized demographic variables: categorical data were reported as frequencies and percentages, and continuous data as medians with interquartile ranges (IQR).

Knowledge scores (0–7) were derived from the number of correct responses. Because data were not normally distributed, non-parametric tests (Wilcoxon rank-sum and Kruskal–Wallis) were used for group comparisons. A multivariable linear regression model identified predictors of higher DDI knowledge, including gender, age, education level, years of experience as a licensed pharmacist, and city of practice. Beta coefficients with 95% confidence intervals (CIs) were reported. For descriptive purposes, scores were categorized as poor (0–2), moderate (3, 4), and good (5–7), consistent with thresholds used in comparable pharmacist DDI knowledge studies. The knowledge score (range 0–7) was treated as a continuous variable in regression analyses, as it represents a composite measure. For simulated patient data, the comparisons were performed using Fisher’s exact test, and *p*-values were adjusted for multiple comparisons using the Benjamini–Hochberg false discovery rate method. Effect sizes were reported to quantify the magnitude of associations. For categorical comparisons assessed using Pearson’s Chi-squared or Fisher’s exact tests, Cramér’s V was calculated. For non-parametric comparisons using the Kruskal–Wallis rank sum test, epsilon-squared (ε^2^) was used as a measure of effect size. Statistical significance was set at *p* < 0.05.

## 3 Results

### 3.1 Demographic characteristics

Among the 356 participating Pharmacists, the majority were male (86.0%), with females accounting for 14.0%. Most respondents were aged 27 to 32 years (43.3%), followed by 33 to 38 years (25.3%). Nearly equal proportions held a Bachelor of Pharmacy (49.2%) or Doctor of Pharmacy (48.6%) degree. The most common city of practice was Makkah (41.6%), followed by Jeddah (36.5%) and Madinah (21.9%). More than half of the participants had 1 to 5 years of professional experience (52.5%, Table 1).

**Table 1:** Demographic characteristics.

Characteristic	Description
Gender	
Male	306 (86.0%)
Female	50 (14.0%)
Age	
21 to 26*	65 (18.3%)
27 to 32	154 (43.3%)
33 to 38	90 (25.3%)
39 or more	47 (13.2%)
Highest pharmacy-related educational qualification	
Bachelor (B. Pharm)	175 (49.2%)
Bachelor (Pharm. D)	173 (48.6%)
Master	6 (1.7%)
Doctor of Philosophy (PhD)	2 (0.6%)
City of current practice	
Jeddah	130 (36.5%)
Madinah	78 (21.9%)
Makkah	148 (41.6%)
Years of experience as a licensed pharmacist	
< 1 year	28 (7.9%)
1 to 5 years	187 (52.5%)
6 to 10 years	70 (19.7%)
More than 10 years	71 (19.9%)

*n* (%).

*Although the age category included 21 years as the lower boundary, all participants in the study were aged ≥23 years.

### 3.2 Participants’ responses to knowledge items regarding drug–drug interactions

Correct knowledge of clinically significant drug–drug interactions varied across scenarios. Most participants correctly identified the interactions between Omeprazole and Clopidogrel (68.5%), Ibuprofen and Furosemide (67.1%), and Phenytoin and Warfarin (65.2%). Similarly, 62.9% recognized the interaction between Fluconazole and Phenytoin. A high percentage (79.2%) correctly indicated no clinically significant interaction between Amoxicillin and Acetaminophen, and 68.3% did the same for Acetaminophen and Celecoxib. However, only 31.2% correctly reported the absence of interaction between Digoxin and Sildenafil (Table 2).

**Table 2:** Participants’ responses to knowledge items regarding drug–drug interactions (*n* = 356).

Characteristic	Description
There is a drug interaction between Ibuprofen and Furosemide	
No – There is no clinically significant interaction between these drugs	50 (14.0%)
Yes – Loop diuretic may reduce the analgesic effect of NSAIDs	37 (10.4%)
Yes – NSAIDs may reduce diuretic efficacy. Loop diuretic may increase NSAIDs-induced nephrotoxicity^*^	239 (67.1%)
Yes – NSAIDs increase the absorption of loop diuretic leading to dehydration	30 (8.4%)
There is a drug interaction between Amoxicillin and Acetaminophen	
No – There is no clinically significant interaction between these drugs^*^	282 (79.2%)
Yes – Interaction affecting absorption or metabolism	23 (6.5%)
Yes – Potential harm or adverse effect to the patient	31 (8.7%)
Yes – Decreased effectiveness of one or both drugs	20 (5.6%)
There is a drug interaction between Fluconazole and Phenytoin	
No – There is no clinically significant interaction between these drugs	13 (3.7%)
Yes – Fluconazole may increase Phenytoin serum levels via CYP2C19 inhibition^*^	224 (62.9%)
Yes – Phenytoin increases the antifungal action of Fluconazole	59 (16.6%)
Yes – Fluconazole reduces the effect of Phenytoin by enhancing its clearance	60 (16.9%)
There is a drug interaction between Digoxin and Sildenafil	
No – There is no clinically significant interaction between these drugs^*^	111 (31.2%)
Yes – Digoxin enhances the hypotensive effect of Sildenafil	74 (20.8%)
Yes – Sildenafil increases the risk of Digoxin-induced arrhythmias	123 (34.6%)
Yes – Digoxin reduces the vasodilatory efficacy of Sildenafil	48 (13.5%)
There is a drug interaction between Phenytoin and Warfarin	
No – There is no clinically significant interaction between these drugs	29 (8.1%)
Yes – Phenytoin may increase or decrease the anticoagulant effect of Warfarin; Warfarin may increase Phenytoin serum levels^*^	232 (65.2%)
Yes – Warfarin reduces Phenytoin absorption causing treatment failure	41 (11.5%)
Yes – Phenytoin completely blocks the action of Warfarin leading to clot formation	54 (15.2%)
There is a drug interaction between Omeprazole and Clopidogrel	
No – There is no clinically significant interaction between these drugs	27 (7.6%)
Yes – Omeprazole may reduce the antiplatelet effect of Clopidogrel by decreasing its active metabolite exposure^*^	244 (68.5%)
Yes – Omeprazole increases bleeding risk by enhancing Clopidogrel action	60 (16.9%)
Yes – Clopidogrel raises stomach PH, reducing Omeprazole absorption	25 (7.0%)
There is a drug interaction between Acetaminophen and Celecoxib	
No – There is no clinically significant interaction between these drugs^*^	243 (68.3%)
Yes – Celecoxib may increase the hepatotoxicity of Acetaminophen due to CYP2C9 inhibition	62 (17.4%)
Yes – Concurrent use may lead to enhanced risk of renal impairment, especially in dehydrated patients	29 (8.1%)
Yes – Acetaminophen may interfere with the anti-inflammatory action of Celecoxib	22 (6.2%)

^*^An asterisk indicates a correct answer.

*n* (%).

### 3.3 Factors and predictors of knowledge scores regarding DDIs

The assumptions of the multiple linear regression model were assessed prior to interpretation of the results. Homoscedasticity was evaluated using the Breusch–Pagan test and was not violated (*p* = 0.237). Independence of errors was examined using the Durbin–Watson statistic (1.72), which indicated no substantial autocorrelation. Multicollinearity was assessed using variance inflation factors (VIF), and no evidence of collinearity was detected (all VIF values < 5). Although the Shapiro–Wilk test suggested a deviation from normality of residuals (*p* < 0.001), visual inspection of the Q–Q plot demonstrated approximate normality, with only minor deviations at the tails. Given the relatively large sample size (*n* = 356), these deviations were not considered to meaningfully affect model validity. Therefore, the assumptions of linear regression were deemed adequately satisfied.

**Figure 1:**
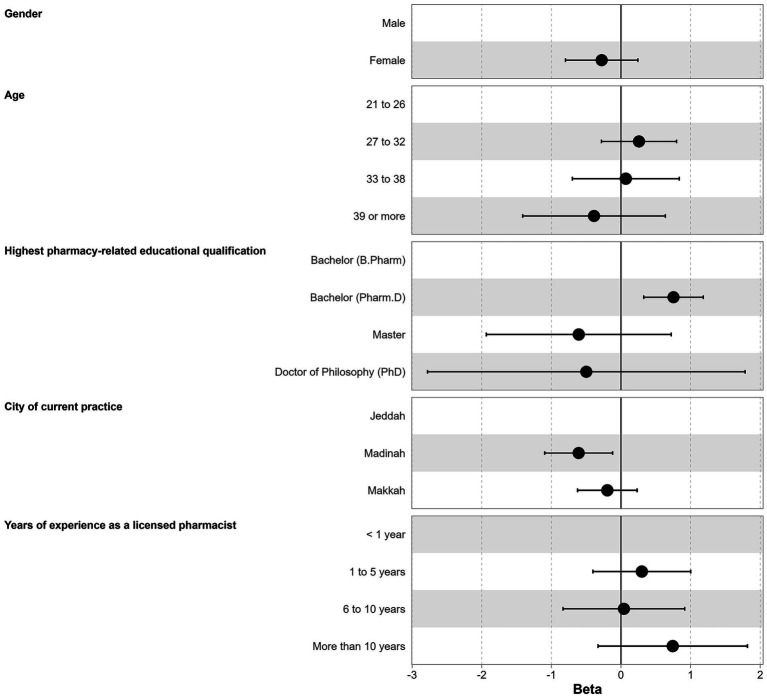
Results of the multivariable linear regression for the predictors of knowledge.

Results showed that higher knowledge scores were significantly associated with educational qualification and city of practice. Participants holding a Pharm. D. degree had significantly higher scores compared to B. Pharm holders (median = 5.0, IQR = 4.0 to 6.0 vs. median = 4.0, IQR = 3.0 to 5.0, beta = 0.75, 95% CI, 0.33 to 1.18, *p* < 0.001). Pharmacists practicing in Madinah had lower scores than those in Jeddah (median = 4.0, IQR = 3.0 to 5.0 vs. median = 5.0, IQR = 4.0 to 6.0, beta = −0.61, 95% CI, −1.09 to −0.12, *p* = 0.015). No significant differences in knowledge scores were found across gender or most age categories (Table 3; Figure 1).

**Table 3:** Factors and predictors of knowledge scores regarding DDIs.

Variable	Inferential analysis		Multivariable regression		
	Median (IQR)	*p*-value	Beta	95% CI	*p*-value
Gender		0.748			
Male	5.0 (3.0, 6.0)		Reference	Reference	
Female	5.0 (3.0, 6.0)		−0.28	−0.80, 0.24	0.296
Age		0.028			
21 to 26	5.0 (3.0, 6.0)		Reference	Reference	
27 to 32	5.0 (3.0, 6.0)		0.26	−0.28, 0.80	0.347
33 to 38	4.0 (3.0, 5.0)		0.07	−0.70, 0.83	0.860
39 or more	4.0 (3.0, 5.0)		−0.39	−1.41, 0.63	0.458
Highest pharmacy-related educational qualification		<0.001			
Bachelor (B. Pharm)	4.0 (3.0, 5.0)		Reference	Reference	
Bachelor (Pharm. D)	5.0 (4.0, 6.0)		0.75	0.33, 1.18	<0.001
Master	3.5 (3.0, 5.0)		−0.61	−1.93, 0.72	0.370
Doctor of Philosophy (PhD)	3.0 (3.0, 3.0)		−0.50	−2.77, 1.78	0.668
City of current practice		<0.001			
Jeddah	5.0 (4.0, 6.0)		Reference	Reference	
Madinah	4.0 (3.0, 5.0)		−0.61	−1.09, −0.12	0.015
Makkah	5.0 (3.0, 6.0)		−0.20	−0.62, 0.23	0.368
Years of experience as a licensed pharmacist		0.045			
< 1 year	5.0 (3.0, 6.0)		Reference	Reference	
1 to 5 years	5.0 (3.0, 6.0)		0.30	−0.40, 1.00	0.403
6 to 10 years	4.0 (3.0, 5.0)		0.04	−0.83, 0.91	0.924
More than 10 years	4.0 (3.0, 5.0)		0.74	−0.33, 1.81	0.174

IQR, interquartile range; CI, confidence interval.

Wilcoxon rank sum test; Kruskal-Wallis rank sum test.

### 3.4 Practice and daily management of drug–drug interactions

More than half of Pharmacists (52.0%) reported always checking for DDIs before dispensing, and 48.0% primarily used electronic websites such as Lexicomp and Micromedex. Weekly (32.9%) and daily (20.5%) encounters with potential DDIs were common. Upon detecting a DDI, 50.0% contacted the prescribing physician, while 25.6% recommended alternatives. A total of 63.2% had encountered clinically significant DDIs at least once. Regarding confidence, 92.4% reported being either somewhat or very confident in identifying DDIs. Most Pharmacists (82.0%) had access to electronic tools, either always (55.9%) or inconsistently (26.1%). The most reported barrier to managing DDIs was poor communication with prescribers (61.8%), followed by lack of time (29.2%). Nearly half received training during pharmacy education (48.3%), and 31.7% had professional development. While 71.6% believed current systems significantly reduce DDI risks, high workload (64.6%) and stress or burnout (50.0%) were common workplace challenges (Table 4).

**Table 4:** Practice and daily management of drug–drug interactions (*n* = 356).

Characteristic	Description
Frequency of encountering potential drug–drug interactions (DDIs)	
Rarely	94 (26.4%)
Monthly	72 (20.2%)
Weekly	117 (32.9%)
Daily	73 (20.5%)
Frequency of checking for DDIs before dispensing	
Always	185 (52.0%)
Often	97 (27.2%)
Sometimes	67 (18.8%)
Never	7 (2.0%)
Primary tool used to detect DDIs	
Electronic website (e.g., Lexicomp, Micromedex, UpToDate)	171 (48.0%)
Mobile applications (e.g., Medscape)	91 (25.6%)
AI-based tools (e.g., ChatGPT, MediSearch, Med-PaLM)	71 (19.9%)
Printed references (e.g., Pharmacology textbooks, Scientific journals)	8 (2.2%)
I do not use any tool	15 (4.2%)
Typical response upon detecting a DDI	
Contact the physician	178 (50.0%)
Recommend an alternative	91 (25.6%)
Dispense and counsel	75 (21.1%)
Do nothing	12 (3.4%)
Ever encountered clinically significant DDIs resulting in patient harm or requiring medical intervention	
No	92 (25.8%)
Yes – once	116 (32.6%)
Yes – more than once	109 (30.6%)
Not sure	39 (11.0%)
Confidence in ability to identify clinically significant DDIs	
Not confident	19 (5.3%)
Somewhat confident	173 (48.6%)
Very confident	156 (43.8%)
Not sure	8 (2.2%)
Access to electronic tools in the current pharmacy setting that automatically detect and flag potential DDIs	
No	54 (15.2%)
Yes, but not consistently	93 (26.1%)
Yes, always	199 (55.9%)
Not sure	10 (2.8%)
Main barriers faced when managing potential DDIs	
No barrier	2 (0.6%)
Poor communication with the prescribers	220 (61.8%)
Lack of time	104 (29.2%)
Limited training or knowledge	29 (8.1%)
All of the above and also the more significant barrier in community pharmacy is the lack of patience of the patients	1 (0.3%)
Ever received formal training specifically focused on identifying and managing DDIs	
No, and I do not think it’s necessary	3 (0.8%)
No, but I would like to	68 (19.1%)
Yes, during pharmacy education	172 (48.3%)
Yes, through professional development or workshops	113 (31.7%)
Perception of whether the current pharmacy system (software/tools) helps reduce the risk of DDIs	
Yes, significantly	255 (71.6%)
Yes, but not enough	101 (28.4%)
No, it does not help	0 (0.0%)
Which of the following workplace-related factors negatively affect your ability to detect and manage DDIs?	
High workload	230 (64.6%)
Long working hours	152 (42.7%)
Low salary	38 (10.7%)
Stress or burnout	178 (50.0%)
Others	5 (1.4%)
Nothing	5 (1.4%)

*n* (%).

### 3.5 Factors associated with the frequency of encountering DDIs

**Figure 2:**
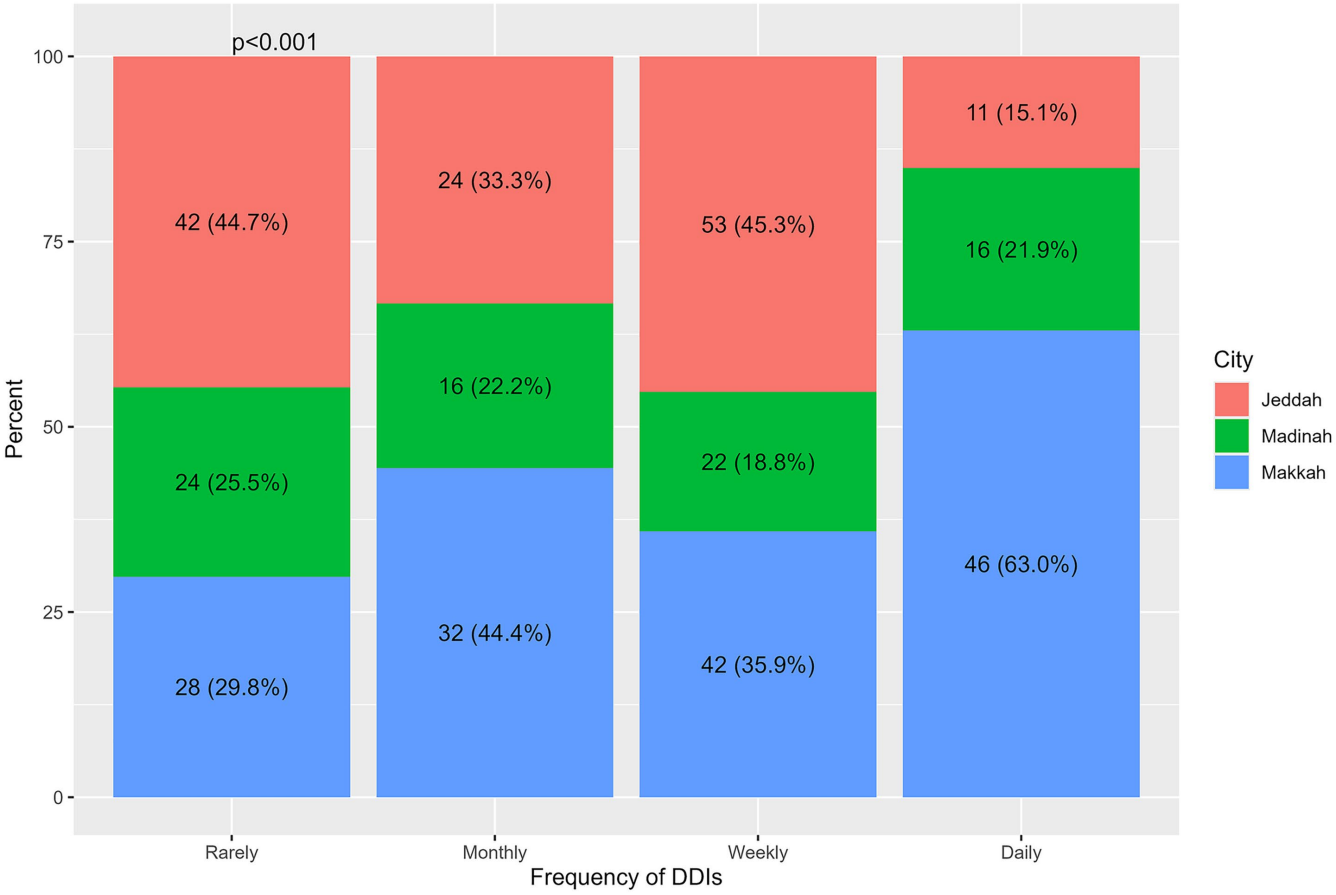
Statistical differences in the frequency of DDIs terms of cities.

Significant variation in the frequency of DDI encounters was observed based on the city of practice (*p* < 0.001). Pharmacists in Makkah reported a higher proportion of daily DDI encounters (63.0%) compared to those in Jeddah (15.1%). No statistically significant differences were found in DDI encounter frequency by gender (*p* = 0.364), age (*p* = 0.694), education level (*p* = 0.156), years of experience as a licensed pharmacist (*p* = 0.060), or knowledge score (*p* = 0.438) (Table 5; Figure 2).

**Table 5:** Factors associated with the frequency of encountering DDIs (*n* = 356).

Characteristic	Frequency of encountering potential DDIs				ES	*p*-value^*^	*q*-value^§^
	Rarely *N* = 94	Monthly *N* = 72	Weekly *N* = 117	Daily *N* = 73			
Gender					0.095	0.364	0.525
Male	84 (89.4%)	64 (88.9%)	99 (84.6%)	59 (80.8%)			
Female	10 (10.6%)	8 (11.1%)	18 (15.4%)	14 (19.2%)			
Age					0.078	0.694	0.694
21 to 26	13 (13.8%)	15 (20.8%)	21 (17.9%)	16 (21.9%)			
27 to 32	37 (39.4%)	33 (45.8%)	53 (45.3%)	31 (42.5%)			
33 to 38	28 (29.8%)	18 (25.0%)	29 (24.8%)	15 (20.5%)			
39 or more	16 (17.0%)	6 (8.3%)	14 (12.0%)	11 (15.1%)			
Highest pharmacy-related educational qualification					0.118	0.156	0.312
Bachelor (B. Pharm)	54 (57.4%)	35 (48.6%)	54 (46.2%)	32 (43.8%)			
Bachelor (Pharm. D)	37 (39.4%)	35 (48.6%)	61 (52.1%)	40 (54.8%)			
Master	3 (3.2%)	0 (0.0%)	2 (1.7%)	1 (1.4%)			
Doctor of Philosophy (PhD)	0 (0.0%)	2 (2.8%)	0 (0.0%)	0 (0.0%)			
City of current practice					0.194	<0.001	0.003
Jeddah	42 (44.7%)	24 (33.3%)	53 (45.3%)	11 (15.1%)			
Madinah	24 (25.5%)	16 (22.2%)	22 (18.8%)	16 (21.9%)			
Makkah	28 (29.8%)	32 (44.4%)	42 (35.9%)	46 (63.0%)			
Years of experience as a licensed pharmacist					0.124	0.060	0.179
< 1 year	11 (11.7%)	6 (8.3%)	7 (6.0%)	4 (5.5%)			
1 to 5 years	39 (41.5%)	44 (61.1%)	66 (56.4%)	38 (52.1%)			
6 to 10 years	16 (17.0%)	15 (20.8%)	21 (17.9%)	18 (24.7%)			
More than 10 years	28 (29.8%)	7 (9.7%)	23 (19.7%)	13 (17.8%)			
Knowledge score	5.0 (3.0–6.0)	5.0 (3.5–6.0)	5.0 (3.0–6.0)	5.0 (3.0–6.0)	−0.001	0.438	0.525

*n* (%); Median (Q1–Q3).

^*^Pearson’s Chi-squared test; Fisher’s exact test; Kruskal-Wallis rank sum test.

^§^False discovery rate correction for multiple testing.

ES, effect size; Cramér’s V for categorical variables and epsilon-squared (ε^2^) for Kruskal–Wallis test, indicating the strength of association.

### 3.6 Pharmacists’ responses to simulated patients

Among the 134 simulated patient visits, the most common city was Jeddah (38.1%), followed by Makkah (28.4%), Madinah (17.9%), and Taif (15.7%). The most frequently presented DDI scenario was Ibuprofen with Furosemide (36.6%), followed by Omeprazole with Clopidogrel (31.3%). In 86.6% of encounters, Pharmacists dispensed the medications without requesting a prescription, and only 14.9% recognized the potential DDI. Most Pharmacists took no action when presented with a DDI (73.1%), while 11.2% dispensed with a warning and 2.2% suggested an alternative. Reference materials were used in only 5.2% of visits. Most Pharmacists did not ask about concomitant medications (83.6%) or medical history (84.3%), and only 13.4% provided clear counseling on DDI risks. Despite potential interactions, both drugs were dispensed together in 84.3% of cases. The review time was less than one minute in 59.0% of visits, and only 9.7% exceeded 3 minutes (Table 6).

**Table 6:** Pharmacists’ responses to simulated patients (*n* = 134).

Characteristic	Description
City	
Jeddah	51 (38.1%)
Makkah	38 (28.4%)
Madinah	24 (17.9%)
Taif	21 (15.7%)
DDI scenarios presented to the pharmacist during the visit	
Ibuprofen + Furosemide	49 (36.6%)
Omeprazole + Clopidogrel	42 (31.3%)
Fluconazole + Phenytoin	26 (19.4%)
Phenytoin + Warfarin	17 (12.7%)
The pharmacist requested a prescription and refused to dispense the medication without it	
No – dispensed without requesting a prescription	116 (86.6%)
Yes – requested a prescription and refused to dispense without it	18 (13.4%)
The pharmacist recognized the potential drug–drug interaction	
No	96 (71.6%)
Yes	20 (14.9%)
Requested a prescription and refused to dispense without it	18 (13.4%)
Action taken by the pharmacist	
Took no action	98 (73.1%)
Dispensed with warning	15 (11.2%)
Refused to dispense	0 (0.0%)
Suggested alternative	3 (2.2%)
Requested a prescription and refused to dispense without it	18 (13.4%)
Use of any reference by the pharmacist during verification	
No	108 (80.6%)
Yes	7 (5.2%)
Requested a prescription and refused to dispense without it	18 (13.4%)
Maybe	1 (0.7%)
The pharmacist asked about other medications being taken	
No	112 (83.6%)
Yes	4 (3.0%)
Requested a prescription and refused to dispense without it	18 (13.4%)
The pharmacist inquired about medical history or chronic conditions	
No	113 (84.3%)
Yes	3 (2.2%)
Requested a prescription and refused to dispense without it	18 (13.4%)
The pharmacist provided clear counseling about the potential risk	
No	98 (73.1%)
Yes	18 (13.4%)
Requested a prescription and refused to dispense without it	18 (13.4%)
Both drugs were dispensed together despite the interaction	
No	3 (2.2%)
Yes	113 (84.3%)
Requested a prescription and refused to dispense without it	18 (13.4%)
Time spent reviewing the request or searching for information (Total time of a visit)	
<1 min	79 (59.0%)
1–3 min	36 (26.9%)
>3 min	13 (9.7%)
No review	6 (4.5%)

*n* (%).

### 3.7 Statistical differences in pharmacist’s responses by DDI scenario

There were significant differences in Pharmacists’ responses depending on the DDI scenario presented. Recognition of the DDI varied significantly, with the highest for Phenytoin and Warfarin (29.4%) and the lowest for Fluconazole and Phenytoin (7.7%) (*p* < 0.001). The type of action taken also varied significantly (*p* = 0.002); for example, warnings were more frequently given for Phenytoin and Warfarin (35.3%) compared to other combinations. Use of reference tools was significantly more common for Phenytoin and Warfarin (29.4%) than for other interactions (*p* < 0.001). Inquiry about other medications (*p* = 0.004), medical history (*p* = 0.006), and counseling (*p* = 0.007) also differed significantly by scenario, with better performance observed for the Phenytoin and Warfarin pair. Dispensing both interacting drugs together occurred in all cases involving Fluconazole with Phenytoin and Phenytoin with Warfarin (100.0%), with significant differences observed between scenarios (*p* = 0.006). Time spent reviewing the request also varied (*p* = 0.019), with longer reviews more common for Omeprazole and Clopidogrel or Phenytoin and Warfarin combinations (Table 7). All statistically significant associations remained significant after adjustment for multiple comparisons using the Benjamini–Hochberg false discovery rate method.

**Table 7:** Statistical differences in pharmacist’s responses to simulated patients in terms of drug combinations offered to the pharmacists (*n* = 134).

Characteristic	Ibuprofen + Furosemide *N* = 49	Omeprazole + Clopidogrel *N* = 42	Fluconazole + Phenytoin *N* = 26	Phenytoin + Warfarin *N* = 17	ES	*p*-value^*^	*q*-value^§^
City					0.243	0.002	0.006
Jeddah	20 (40.8%)	10 (23.8%)	12 (46.2%)	9 (52.9%)			
Makkah	10 (20.4%)	10 (23.8%)	10 (38.5%)	8 (47.1%)			
Madinah	10 (20.4%)	10 (23.8%)	4 (15.4%)	0 (0.0%)			
Taif	9 (18.4%)	12 (28.6%)	0 (0.0%)	0 (0.0%)			
The pharmacist requested a prescription and refused to dispense the medication without it.					0.183	0.227	0.227
No – dispensed without requesting a prescription	45 (91.8%)	33 (78.6%)	24 (92.3%)	14 (82.4%)			
Yes – requested a prescription and refused to dispense without it	4 (8.2%)	9 (21.4%)	2 (7.7%)	3 (17.6%)			
The pharmacist recognized the potential drug–drug interaction.					0.375	<0.001	<0.001
No	38 (77.6%)	29 (69.0%)	24 (92.3%)	5 (29.4%)			
Yes	4 (8.2%)	11 (26.2%)	2 (7.7%)	3 (17.6%)			
Requested a prescription and refused to dispense without it	7 (14.3%)	2 (4.8%)	0 (0.0%)	9 (52.9%)			
Action taken by the pharmacist.					0.224	0.002	0.006
Took no action	36 (73.5%)	29 (69.0%)	22 (84.6%)	11 (64.7%)			
Dispensed with warning	2 (4.1%)	3 (7.1%)	4 (15.4%)	6 (35.3%)			
Refused to dispense	0 (0.0%)	0 (0.0%)	0 (0.0%)	0 (0.0%)			
Suggested alternative	3 (6.1%)	0 (0.0%)	0 (0.0%)	0 (0.0%)			
Requested a prescription and refused to dispense without it	8 (16.3%)	10 (23.8%)	0 (0.0%)	0 (0.0%)			
Use of any reference by the pharmacist during verification.					0.297	<0.001	<0.001
No	40 (81.6%)	30 (71.4%)	26 (100.0%)	12 (70.6%)			
Yes	1 (2.0%)	1 (2.4%)	0 (0.0%)	5 (29.4%)			
Requested a prescription and refused to dispense without it	8 (16.3%)	10 (23.8%)	0 (0.0%)	0 (0.0%)			
Maybe	0 (0.0%)	1 (2.4%)	0 (0.0%)	0 (0.0%)			
The pharmacist asked about other medications being taken.					0.244	0.004	0.008
No	41 (83.7%)	29 (69.0%)	25 (96.2%)	17 (100.0%)			
Yes	0 (0.0%)	3 (7.1%)	1 (3.8%)	0 (0.0%)			
Requested a prescription and refused to dispense without it	8 (16.3%)	10 (23.8%)	0 (0.0%)	0 (0.0%)			
The pharmacist inquired about medical history or chronic conditions.					0.241	0.006	0.008
No	41 (83.7%)	31 (73.8%)	24 (92.3%)	17 (100.0%)			
Yes	0 (0.0%)	1 (2.4%)	2 (7.7%)	0 (0.0%)			
Requested a prescription and refused to dispense without it	8 (16.3%)	10 (23.8%)	0 (0.0%)	0 (0.0%)			
The pharmacist provided clear counseling about the potential risk.					0.257	0.007	0.009
No	36 (73.5%)	28 (66.7%)	23 (88.5%)	11 (64.7%)			
Yes	5 (10.2%)	4 (9.5%)	3 (11.5%)	6 (35.3%)			
Requested a prescription and refused to dispense without it	8 (16.3%)	10 (23.8%)	0 (0.0%)	0 (0.0%)			
Both drugs were dispensed together despite the interaction.					0.248	0.006	0.008
No	3 (6.1%)	0 (0.0%)	0 (0.0%)	0 (0.0%)			
Yes	38 (77.6%)	32 (76.2%)	26 (100.0%)	17 (100.0%)			
Requested a prescription and refused to dispense without it	8 (16.3%)	10 (23.8%)	0 (0.0%)	0 (0.0%)			
Time spent reviewing the request or searching for information (Total time of a visit)					0.209	0.019	0.022
< 1 min	38 (77.6%)	19 (45.2%)	14 (53.8%)	8 (47.1%)			
1–3 min	5 (10.2%)	16 (38.1%)	9 (34.6%)	6 (35.3%)			
> 3 min	3 (6.1%)	4 (9.5%)	3 (11.5%)	3 (17.6%)			
No review	3 (6.1%)	3 (7.1%)	0 (0.0%)	0 (0.0%)			

*n* (%).

^*^Fisher’s exact test.

^§^False discovery rate correction for multiple testing.

Effect size (Cramér’s V), indicating the strength of association between variables.

## 4 Discussion

This study examined community Pharmacists’ knowledge and real-world management of drug–drug interactions in Western Saudi Arabia through a mixed-methods approach. The findings reveal a consistent pattern: Pharmacists demonstrate moderate theoretical knowledge but exhibit limited translation into practice, a phenomenon with significant implications for patient safety.

### 4.1 Knowledge levels and educational predictors

Pharmacists showed acceptable but suboptimal knowledge levels. Most correctly identified well-established interactions, consistent with previous national studies reporting moderate DDI awareness among Saudi Pharmacists (13, 14, 22, 23). The superior performance of Pharm. D. graduates compared to B. Pharm. holders represents a robust finding that aligns with international literature indicating that Pharm. D. curricula provide stronger grounding in clinical pharmacology and evidence-based practice (24, 25). Pharm. D. programs typically incorporate extensive hospital rotations, clinical case analyses, and therapeutic decision-making exercises, likely enhancing graduates’ ability to identify and manage DDIs in real-world settings (24, 25).

Similar findings have been reported internationally. Studies from Europe, North America, and Southeast Asia have consistently shown that community pharmacists demonstrate reasonable theoretical knowledge of DDIs, particularly for commonly encountered drug pairs, yet gaps persist for clinically complex interactions (7–10). These international observations reinforce the notion that educational structure plays a significant role in shaping pharmacists’ baseline DDI knowledge, while also highlighting that knowledge alone may be insufficient to ensure safe practice.

### 4.2 Regional variations

Significant regional variation was observed, with community Pharmacists in Madinah demonstrating lower knowledge scores compared to Jeddah counterparts. Jeddah, as a larger metropolitan center with high hospital density and academic institutions, likely provides greater exposure to complex cases and continuing professional development opportunities (26). This suggests potential inequity in continuing education resources across regions, emphasizing the need for targeted educational programs in secondary cities to standardize professional competence nationwide.

Pharmacists in Makkah reported significantly higher daily exposure to DDIs compared with colleagues in other cities. This likely reflects Makkah’s unique healthcare context, particularly during Hajj and Umrah seasons when the city experiences an influx of millions of pilgrims with complex therapeutic combinations (27, 28). However, higher exposure frequency did not translate into better DDI detection rates during SP visits, suggesting that exposure alone, without adequate time and resources, may not enhance clinical performance (29).

### 4.3 The knowledge-practice gap

The simulated patient phase revealed a profound gap between perceived competence and real-world performance. Only 14.9% of Pharmacists identified potential DDIs during SP encounters, and most failed to provide adequate counseling or verify interactions through reference tools. Such discrepancies between self-reported and observed practice have been documented in SP-based studies across various healthcare settings (30, 31).

This knowledge-practice gap can be understood through multiple perspectives. From a cognitive psychology viewpoint, the gap may reflect differences between declarative knowledge (knowing that) and procedural knowledge (knowing how to apply it under pressure) (32). Surveys primarily assess declarative knowledge, while actual practice requires integration under time pressure and cognitive load. Behavioral economics offers another lens through concepts such as cognitive biases in high-volume settings, Pharmacists may rely on cognitive shortcuts that, while efficient, can lead to systematic errors when dangerous DDIs are present (33, 34).

Findings from the current study support previous evidence that organizational barriers play a major role in widening the knowledge–practice gap. Consistent with earlier studies, SP encounters were typically brief, often under one minute, limiting opportunities for medication review or use of drug references. High workload and inadequate staffing further restrict Pharmacists’ ability to apply their knowledge effectively (35).

Social and professional factors further complicate the picture. Hierarchical healthcare structures may discourage Pharmacists from questioning physician prescriptions, particularly when communication channels are inadequate (36, 37). The present study identified poor communication with prescribers as the most frequently cited barrier, a finding consistent with broader literature documenting interprofessional communication challenges as major obstacles to pharmaceutical care delivery (38, 39).

### 4.4 Implications and recommendations

Importantly, the challenges identified in this study mirror those reported internationally, indicating that the need for system-level interventions extends beyond the Saudi context and reflects a global patient safety concern. The findings challenge traditional assumptions about pharmacy education’s role in preparing practitioners for safe medication management. While formal education clearly matters, education alone appears insufficient to ensure appropriate practice behaviors. This suggests that pharmacy curricula should extend beyond knowledge transmission to address behavioral, communication, and systems-thinking competencies required for effective practice (40).

System-level interventions are essential. Clinical decision support systems (CDSS) integrated into pharmacy workflow represent one intervention with substantial evidence supporting effectiveness (41, 42). When designed appropriately, with attention to alert specificity and workflow integration, CDSS can significantly reduce missed DDIs (43). However, CDSS implementation faces challenges including alert fatigue and integration difficulties (43).

Staffing levels and workload management constitute another critical systemic factor. Evidence clearly demonstrates that adequate staffing improves safety outcomes (44). Regulatory standards for Pharmacist-to-technician ratios and maximum prescription volumes may be necessary to create conditions where thorough medication review becomes feasible.

Reimbursement and regulatory reforms that explicitly recognize and compensate Pharmacists for clinical services could fundamentally reshape community pharmacy practice. When Pharmacists are financially rewarded for catching DDIs and collaborating with prescribers, practice behaviors align more closely with patient safety priorities (45).

### 4.5 Strengths and limitations

Several strengths of this study merit acknowledgment. First, the sequential explanatory mixed methods design, which integrates survey based knowledge assessment with covert simulated patient observations, provides a more comprehensive and ecologically valid evaluation than either method alone. Second, the multi city sampling across four major cities in Western Saudi Arabia enhances geographic representativeness within the study region. Third, the use of unannounced simulated patient visits, a gold standard observational methodology, minimizes social desirability bias inherent in self report instruments. Fourth, the inclusion of both pharmacokinetic and pharmacodynamic drug-drug interaction scenarios strengthens the content validity of the observational phase. Finally, the large survey sample of 356 participants, together with a structured and expert validated simulated patient checklist, contributes to the overall robustness of the findings.

Several limitations of this study warrant acknowledgment. First, the research was conducted exclusively in community pharmacies within the Western Region of Saudi Arabia, which may limit the generalizability of the findings to other regions or healthcare settings. Second, the simulated patient (SP) visits represented single encounters, which may not fully capture Pharmacists’ consistent or long-term behavior in routine practice. Third, the number of SP visits (134 encounters) was relatively smaller than the number of survey respondents (356 participants), potentially affecting the representativeness of the observational data. Future studies could expand the sample size and geographic coverage, include multiple SP visits per pharmacy, and explore longitudinal or interventional designs to provide a more comprehensive understanding of how Pharmacists manage drug–drug interactions over time. Fourth, the use of convenience sampling for pharmacist recruitment may introduce selection bias and restrict representativeness with respect to the broader population of community pharmacists in the region; findings should therefore be interpreted with appropriate caution. Fifth, the covert nature of the simulated patient visits precluded the use of audio or video recording, introducing potential observer or recall bias; although immediate post-encounter documentation was required as standard protocol, the absence of objective verification is a limitation shared by most covert simulated patient studies in pharmacy practice research.

## 5 Conclusion

Community Pharmacists in Western Saudi Arabia exhibit reasonable theoretical awareness of major drug–drug interactions but demonstrate inconsistent and often inadequate application in real-world practice. The substantial gap between what Pharmacists know and what they do represents a significant patient safety concern that cannot be addressed through educational interventions alone. Regional differences, educational background, and contextual factors all influence DDI management behaviors. Effective DDI management requires not merely individual Pharmacist competence but rather a supportive ecosystem encompassing adequate staffing, efficient clinical decision support systems, robust interprofessional communication channels, and organizational cultures that prioritize patient safety. Bridging the knowledge-practice gap will require coordinated efforts from pharmacy educators, healthcare administrators, policymakers, prescribers, and Pharmacists themselves.

## Data Availability

The original contributions presented in the study are included in the article/Supplementary material, further inquiries can be directed to the corresponding author.
